# The association between adverse childhood experiences and adult cardiac function in the UK Biobank

**DOI:** 10.1093/ehjimp/qyae139

**Published:** 2024-12-19

**Authors:** Juan C Quiroz, Jackie Cooper, Celeste McCracken, Mohammed Y Khanji, Liliana Laranjo, Nay Aung, Aaron Mark Lee, Judit Simon, Theodore Murphy, Luca Biasiolli, Stefan K Piechnik, Pal Maurovich-Horvat, Steffen E Petersen, Zahra Raisi-Estabragh

**Affiliations:** Centre for Big Data Research in Health (CBDRH), The University of New South Wales (UNSW), Level 2, AGSM Building G27, Botany St, Kensington, NSW 2033, Australia; William Harvey Research Institute, NIHR Barts Biomedical Research Centre, Queen Mary University of London, Charterhouse Square, London EC1M 6BQ, UK; William Harvey Research Institute, NIHR Barts Biomedical Research Centre, Queen Mary University of London, Charterhouse Square, London EC1M 6BQ, UK; Division of Cardiovascular Medicine, Radcliffe Department of Medicine, University of Oxford, National Institute for Health Research Oxford Biomedical Research Centre, Oxford OX3 9DU, UK; William Harvey Research Institute, NIHR Barts Biomedical Research Centre, Queen Mary University of London, Charterhouse Square, London EC1M 6BQ, UK; Barts Heart Centre, St Bartholomew’s Hospital, Barts Health NHS Trust, West Smithfield, London EC1A 7BE, UK; Faculty of Medicine and Health, Westmead Applied Research Centre (WARC), Sydney Medical School, The University of Sydney, Westmead, Australia; William Harvey Research Institute, NIHR Barts Biomedical Research Centre, Queen Mary University of London, Charterhouse Square, London EC1M 6BQ, UK; Barts Heart Centre, St Bartholomew’s Hospital, Barts Health NHS Trust, West Smithfield, London EC1A 7BE, UK; William Harvey Research Institute, NIHR Barts Biomedical Research Centre, Queen Mary University of London, Charterhouse Square, London EC1M 6BQ, UK; MTA-SE Cardiovascular Imaging Research Group, Heart and Vascular Center, Semmelweis University, Budapest, Hungary; Department of Cardiology and Cardiovascular Imaging, Beacon Hospital, Dublin, Ireland; Division of Cardiovascular Medicine, Radcliffe Department of Medicine, University of Oxford, National Institute for Health Research Oxford Biomedical Research Centre, Oxford OX3 9DU, UK; Division of Cardiovascular Medicine, Radcliffe Department of Medicine, University of Oxford, National Institute for Health Research Oxford Biomedical Research Centre, Oxford OX3 9DU, UK; MTA-SE Cardiovascular Imaging Research Group, Heart and Vascular Center, Semmelweis University, Budapest, Hungary; William Harvey Research Institute, NIHR Barts Biomedical Research Centre, Queen Mary University of London, Charterhouse Square, London EC1M 6BQ, UK; Barts Heart Centre, St Bartholomew’s Hospital, Barts Health NHS Trust, West Smithfield, London EC1A 7BE, UK; Health Data Research UK, London, UK; Alan Turing Institute, London, UK; William Harvey Research Institute, NIHR Barts Biomedical Research Centre, Queen Mary University of London, Charterhouse Square, London EC1M 6BQ, UK; Barts Heart Centre, St Bartholomew’s Hospital, Barts Health NHS Trust, West Smithfield, London EC1A 7BE, UK

**Keywords:** cardiovascular imaging phenotypes, cardiovascular magnetic resonance, childhood maltreatment, child abuse, neglect, cardiac remodelling

## Abstract

**Aims:**

The importance of early life factors in determining health in later adulthood is increasingly recognized. This study evaluated the association of adverse childhood experiences (ACEs) with cardiovascular magnetic resonance (CMR) phenotypes.

**Methods and results:**

UK Biobank participants who had completed CMR and the self-reported questionnaire on traumatic childhood experiences were included. Images were analysed using automated pipelines to extract measures of left and right ventricular (LV and RV) structure and function, myocardial character, and arterial compliance. Multivariable linear regression was used to estimate the association of childhood adversity with CMR phenotypes adjusting for age, sex, deprivation, education, obesity, smoking, alcohol intake, exercise level, diabetes, hypertension, and hypercholesterolaemia. Amongst 30 814 participants analysed, 6023 (19.5%) experienced physical abuse, 2746 (8.9%) sexual abuse, 4685 (15.2%) emotional abuse, 6822 (22.1%) emotional neglect, and 4534 (14.7%) physical neglect. Except for physical abuse, women reported greater rates of childhood adversity than men. Collectively, all types of childhood adversity were associated with smaller LV and RV volumes, greater LV mass, a concentric pattern of LV remodelling, poorer LV and RV function, lower aortic compliance, and greater arterial stiffness. Sexual abuse was associated with unhealthy CMR phenotypes in age- and sex-adjusted models, but these relationships were attenuated in fully adjusted models. Physical neglect had the most prominent pattern of adverse cardiovascular remodelling.

**Conclusion:**

ACEs were associated with unhealthy cardiovascular remodelling in adulthood, independent of traditional cardiovascular risk factors. These findings support the consideration of early life factors in cardiovascular disease risk assessment.

## Graphical Abstract

**Graphical Abstract:**
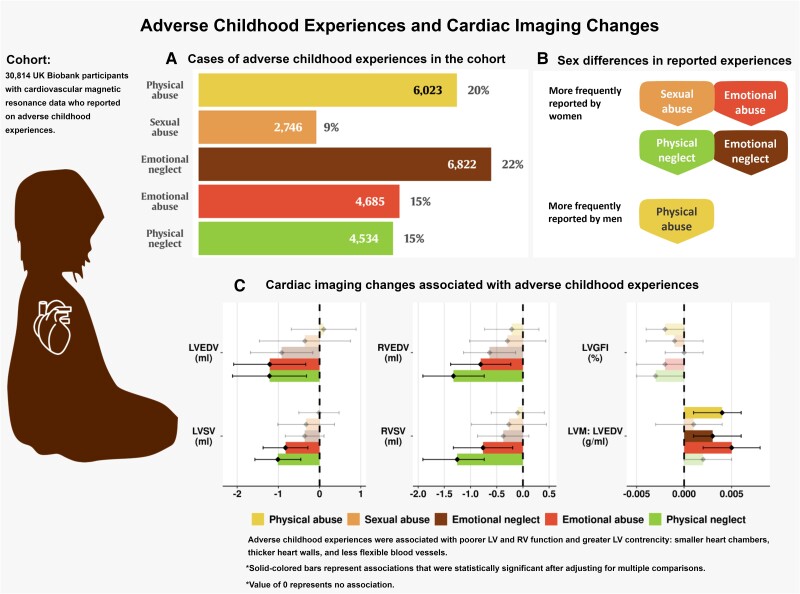
Associations of adverse childhood experiences (ACEs) with cardiac function and structure. ACEs were associated with smaller left ventricular (LV) and right ventricular (RV) volumes, a concentric pattern of LV remodelling, and poorer LV and RV function. Except for physical abuse, women reported greater rates of childhood adversity than men. LVEDV, left ventricular end-diastolic volume; LVSV, left ventricular stroke volume; LVGFI, left ventricular global functional index; RVEDV, right ventricular end-diastolic volume; RVSV, right ventricular stroke volume.

## Introduction

Adverse experiences and trauma in early life have a profound effect on physical and mental well-being. Existing work demonstrates links between adverse childhood experiences (ACEs) and increased health risks in later adulthood,^1–5^ including increased incidence of cardiovascular diseases (CVDs) (e.g. ischaemic heart disease, stroke, and cerebrovascular disease)^6–8^ and poorer cardiovascular outcomes.^9,10^ In light of the growing evidence in this area, the American Heart Association has highlighted the impact of childhood adversity on later cardiovascular health in a dedicated Scientific Statement.^11^

The underlying biological pathways linking childhood adversity with CVD in adulthood are not well understood. Postulated mechanisms include a pro-inflammatory profile, chronic physiologic stress, poor mental health, and greater propensity to cardiometabolic diseases.^6,10,12^ Adversity in early life is also closely linked to socio-economic deprivation, which can impact life chances and health outcomes throughout the entire life course.^13,14^ Since the cardiovascular consequences of these early life events typically manifest long after the incidence of childhood adversity,^11^ detection of subclinical measures is critical to early risk stratification and for initiation of targeted preventive interventions before disease occurrence.

The main body of evidence in the field is focused on links between childhood adversity and the incidence of clinical CVD.^6,8,10^ A smaller number of studies report associations of ACEs with subclinical measures of poorer cardiovascular health in adulthood, including increased carotid artery intima-media thickness (IMT)^15^ and greater systemic inflammation [C-reactive protein, interleukin (IL)-6, fibrinogen, E-selectin, and ICAM-1].^5,16,17^ Greater IMT is a marker for early atherosclerosis,^18,19^ and systemic inflammation is associated with negative myocardial structure and function and CVD.^5,20,21^ ACEs have also been linked to subclinical markers of poor cardiovascular health in adolescents and young adults, including greater arterial stiffness,^22^ circulating endothelin-1, diastolic blood pressure, total peripheral resistance, and pulse wave velocity.^23^

Cardiovascular imaging can provide a reliable capture of subclinical alterations at a whole organ level. Cardiovascular magnetic resonance (CMR) is the reference modality for assessing cardiovascular structure and function, with CMR-derived phenotypes providing sensitive subclinical indicators of cardiovascular health. In this study, we examined previously unreported associations of childhood adversity with CMR measures of cardiovascular structure and function in over 30 000 middle-aged adults from the UK Biobank. This work aims to provide new insights into the potential impact of a range of ACEs on cardiovascular health in adulthood.

## Methods

### Setting and study population

The UK Biobank cohort recruited over 500 000 individuals aged 40–69 years across the UK from 2006 to 2010. Participants underwent detailed baseline characterization according to a pre-defined research protocol.^24^ Individuals who were unable to complete the UK Biobank baseline assessment due to discomfort or ill health, or individuals who were not able to provide informed consent, were not recruited. The UK Biobank Imaging Study (2015–ongoing) aims to perform multi-organ imaging for a 20% subset of the UK Biobank cohort, including a detailed CMR protocol. Our study included participants with available CMR data who completed the questions regarding traumatic childhood experiences^25^ as part of the UK Biobank online follow-up questionnaire.

### Adverse childhood experiences

The following types of childhood maltreatment were considered as the exposures of interest: physical abuse, sexual abuse, emotional abuse, emotional neglect, and physical neglect. These were defined from self-reported answers to questions on traumatic experiences during childhood: felt hated by a family member as a child (emotional abuse), physically abused by family as a child (physical abuse), felt loved as a child (emotional neglect), sexually molested as a child (sexual abuse), and someone available to take the child to the doctor (physical neglect). Following the coding procedure used in prior studies of childhood adversity in the UK Biobank,^26^ we converted these exposure variables into binary fields indicating whether the participant experienced the event. The specific thresholds used for each exposure are detailed in the Supplementary Materials.

### Image acquisition and analysis

The UK Biobank Imaging study used standardized CMR protocols with uniform equipment (1.5T MAGNETOM Aera, Siemens) and staff training.^27^ Ventricular function was assessed using long-axis images and a short-axis stack covering both ventricles from base to apex. Myocardial tissue character was assessed using myocardial native T1 mapping in one mid-ventricular short-axis slice. Arterial compliance was estimated using aortic distensibility (AoD) measured from transverse cuts of the thoracic aorta. Image acquisition and analysis details are included in the Supplementary Materials.

We included the following CMR metrics as the outcomes of interest: left ventricular (LV) end-diastolic volume (LVEDV), LV stroke volume (LVSV), LV mass (LVM), LVM/LVEDV ratio, LV global longitudinal strain, LV global functional index (GFI), myocardial native T1, AoD, right ventricular (RV) end-diastolic volume (RVEDV), and RV stroke volume (RVSV). We included the arterial stiffness index (ASI) derived from finger plethysmography as an additional indicator of arterial health, measured as part of standard UK Biobank protocols.^28^

### Statistical analysis

Statistical analysis was performed using R 4.1.0. Summary statistics were presented as mean and standard deviation or median and interquartile range for continuous variables and percentages with frequency for categorical data. We compared the proportions of men and women who experienced each exposure with a two-sample *z*-test.

Multivariable linear regression was used to examine the association of each exposure variable with individual CMR metrics. We included participants with CMR data and at least one adverse childhood exposure. We first present associations in minimally adjusted (age and sex) models. Fully adjusted models included additional variables that are traditional cardiovascular risk factors and that have been used in prior studies to report the association of CMR imaging phenotypes in the UK Biobank and cardiac outcomes^29^: age, sex, body mass index (BMI), Townsend deprivation score, smoking, diabetes, hypertension, hypercholesterolaemia, alcohol use, exercise, and education. Age, self-reported sex, alcohol intake frequency, smoking status (current vs. never/previous), and physical activity were recorded at the UK Biobank imaging visit. BMI was calculated from height and weight measurements taken at imaging. Townsend deprivation index,^30^ a postcode-based measure of socio-economic deprivation, and self-reported educational attainment were recorded at baseline. Answers to exercise questions were converted into summed metabolic equivalent task-minutes per week as per published guidance for the International Physical Activity Questionnaire.^31^ Diabetes, hypertension, and hypercholesterolaemia were ascertained at imaging based on both self-reported diagnoses and evidence from linked health records.

Residual plots were used to check for heteroscedasticity and extreme observations, with extreme observations removed. The AoD and RVEDV metrics were log-transformed to reduce heteroscedasticity. We report the association of each exposure with absolute change in CMR measure with corresponding 95% confidence intervals (CIs) and *P*-values. For log-transformed variables, we report percentage change. To correct for multiple comparisons for multiple hypotheses, a false discovery rate (FDR) adjusted *P*-value (*q*-value) was included in the analysis. FDR *q*-values <0.05 were considered statistically significant. We tested for interaction effects by sex by adding a sex–exposure interaction term to each of the fully adjusted models, with females as the reference group.

## Results

### Baseline characteristics

The analysis included 30 814 participants with CMR data and answers to at least one of the ACEs questions available (*Table 1*). The average age was 64.1 ± 7.7 years, and there were slightly more women (53.3%; *n* = 16 428). The rates of smoking, diabetes, hypertension, and hypercholesterolaemia were 3.3% (*n* = 997), 5.3% (*n* = 1648), 27.8% (*n* = 8563), and 24.7% (*n* = 7626), respectively. Overall, the study sample was less deprived (or more affluent, median Townsend = −2.61) than the general UK population (2011 census median Townsend = −0.35).

**Table 1:** Baseline participant characteristics

	Whole cohort	Physical abuse	Sexual abuse	Emotional abuse	Emotional neglect	Physical neglect
*N*	30 814	6023	2746	4685	6822	4534
Age (years)^a^	64.1 ± 7.7	62.9 ± 7.7	63.7 ± 7.6	62.4 ± 7.7	63.7 ± 7.6	65.5 ± 7.7
Women	53.3% (16 428)	49.2% (2961)	69.7% (1915)	61.0% (2860)	55.3% (3773)	56.7% (2571)
Current smoker	3.3% (997)	4.5% (271)	3.9% (105)	5.0% (232)	4.3% (293)	3.4% (155)
Diabetes	5.3% (1648)	6.3% (377)	6.5% (178)	5.8% (272)	6.2% (423)	7.3% (333)
Hypertension	27.8% (8563)	28.5% (1715)	28.1% (772)	28.5% (1335)	29.0% (1978)	32.2% (1459)
Hypercholesterolaemia	24.7% (7626)	23.9% (1437)	22.9% (630)	22.3% (1044)	24.5% (1671)	28.0% (1268)
Alcohol intake ≥3 times/week	45.8% (14 017)	43.9% (2631)	41.3% (1126)	42.1% (1962)	42.7% (2913)	40.4% (1817)
BMI (kg/m^2^)^a^	26.3 ± 4.4	27.1 ± 4.7	26.8 ± 4.7	26.9 ± 4.8	26.7 ± 4.6	26.8 ± 4.5
Townsend deprivation score^b^	−2.61 [−3.88, −0.46]	−2.38 [−3.82, −0.01]	−2.19 [−3.67, 0.43]	−2.08 [−3.65, 0.53]	−2.25 [−3.74, 0.27]	−2.46 [−3.89, −0.12]
IPAQ score (METs/week)^b^	2148 [1113, 3822]	2147 [1094, 3929]	2106 [1050, 3759]	2133 [1044, 3866]	2076 [1040, 3786]	2228 [1110, 4068]
Degree or professional qualification	68.7% (21188)	67.8% (4080)	69.5% (1909)	70.1% (3284)	65.7% (4482)	58.1% (2631)

IPAQ, International Physical Activity Questionnaire; MET, metabolic equivalent of task.

^a^Mean ± standard deviation.

^b^Median [25th percentile, 75th percentile].

### Physical abuse

A history of childhood physical abuse was reported by 6023 (19.5%) participants, with men reporting physical abuse at a greater rate than women (21.3% vs. 18.0%, as detailed in Supplementary data online, *Table S1*). Individuals with a history of physical abuse had a slightly poorer cardiometabolic profile and had higher rates of smoking than the whole cohort (*Table 1*).

In minimally adjusted models (full results presented in Supplementary data online, *Table S2*), childhood physical abuse was related to significantly larger LV end-diastolic volumes (higher LVEDV), greater LV mass (higher LVM), greater LV concentricity (higher LVM/LVEDV), and higher arterial stiffness (higher ASI). In models including additional adjustment for potential cardiometabolic and lifestyle mediators, physical abuse was linked to greater LV concentricity (*Table 2*), with a trend towards significance for larger LVM. There was a significant interaction between sex and physical abuse for LVM, further explored in Supplementary data online, *Table S3*. Physical abuse was associated with greater LVM in men [*β* = 1.57, 95% CI (0.98, 2.17), *P* < 0.001], but not in women [*β* = −0.54, 95% CI (−1.15, 0.06), *P* = 0.08]. There was no significant interaction between sex and physical abuse for all other metrics.

**Table 2:** Associations of ACEs with CMR metrics

	Physical abuse	Sexual abuse	Emotional neglect	Emotional abuse	Physical neglect
LVEDV (mL)	0.101 (−0.685, 0.886)	−0.356 (−1.463, 0.751)	−0.921 (−1.678, −0.164)	**−1.210** **(−2.085, −0.335)**	**−1.216** **(−2.113, −0.318)**
	*q* = 0.882	*q* = 0.677	*q* = 0.067	***q* = 0.041**	***q* = 0.042**
LVSV (mL)	−0.015 (−0.503, 0.474)	−0.327 (−1.015, 0.361)	−0.363 (−0.834, 0.108)	**−0.827** **(−1.371, −0.283)**	**−1.013** **(−1.571, −0.455)**
	*q* = 0.960	*q* = 0.569	*q* = 0.279	***q* = 0.028**	***q* = 0.005**
LVM (g)	0.538 (0.114, 0.963)	−0.142 (−0.740, 0.457)	−0.029 (−0.438, 0.380)	−0.012 (−0.485, 0.461)	−0.480 (−0.966, 0.005)
	*q* = 0.055	*q* = 0.804	*q* = 0.941	*q* = 0.960	*q* = 0.170
LVM: LVEDV (g/mL)	**0.004** **(0.001, 0.006)**	0.001 (−0.003, 0.004)	**0.003** **(0.001, 0.006)**	**0.005** **(0.002, 0.008)**	0.002 (0.000, 0.005)
	***q* = 0.028**	*q* = 0.829	***q* = 0.031**	***q* = 0.005**	*q* = 0.249
LVGLS (%)	0.050 (−0.053, 0.154)	0.063 (−0.084, 0.211)	0.016 (−0.083, 0.116)	0.050 (−0.065, 0.165)	−0.018 (−0.137, 0.100)
	*q* = 0.569	*q* = 0.595	*q* = 0.874	*q* = 0.595	*q* = 0.874
LVGFI (%)	−0.002 (−0.004, 0.000)	−0.001 (−0.004, 0.002)	0.000 (−0.002, 0.002)	−0.002 (−0.005, 0.000)	−0.003 (−0.005, 0.000)
	*q* = 0.179	*q* = 0.615	*q* = 0.882	*q* = 0.179	*q* = 0.099
T1 (ms)	0.814 (−0.220, 1.848)	0.048 (−1.412, 1.508)	0.812 (−0.187, 1.812)	1.378 (0.224, 2.532)	0.997 (−0.189, 2.183)
	*q* = 0.279	*q* = 0.960	*q* = 0.265	*q* = 0.071	*q* = 0.249
AoD^a^ (×10^−3^ mmHg)	1.363 (−0.475, 3.236)	2.484 (−0.156, 5.193)	−0.958 (−2.690, 0.804)	0.702 (−1.333, 2.779)	−1.233 (−3.283, 0.860)
	*q* = 0.299	*q* = 0.179	*q* = 0.521	*q* = 0.657	*q* = 0.478
ASI (m/s)	0.055 (−0.040, 0.150)	−0.070 (−0.205, 0.065)	−0.044 (−0.135, 0.048)	−0.046 (−0.152, 0.061)	0.012 (−0.096, 0.120)
	*q* = 0.478	*q* = 0.550	*q* = 0.569	*q* = 0.595	*q* = 0.891
RVEDV^a^ (mL)	−0.214 (−0.731, 0.305)	−0.295 (−1.021, 0.437)	−0.638 (−1.134, −0.140)	**−0.808** **(−1.380, −0.232)**	**−1.326** **(−1.910, −0.739)**
	*q* = 0.605	*q* = 0.605	*q* = 0.055	***q* = 0.041**	***q* < 0.001**
RVSV (mL)	−0.099 (−0.607, 0.409)	−0.268 (−0.983, 0.448)	−0.376 (−0.866, 0.113)	**−0.762** **(−1.328, −0.196)**	**−1.254** **(−1.910, −0.739)**
	*q* = 0.841	*q* = 0.621	*q* = 0.279	***q* = 0.042**	***q* < 0.001**

Results are absolute changes in CMR metric expressed as Beta (95% CI) and FDR *q*-value below. The association of each exposure (binary Yes/No) with CMR metrics was estimated with linear regression models adjusted for age, sex, BMI, Townsend deprivation score, smoking, diabetes, hypertension, hypercholesterolaemia, alcohol use, exercise, and education. Bold values denote statistical significance after adjustment for multiple comparisons (FDR *q*-value < 0.05).

LVEDV, left ventricular end-diastolic volume; LVSV, left ventricular stroke volume; LVM, left ventricular mass; LVGLS, left ventricular global longitudinal strain; LVGFI, left ventricular global functional index; AoD, aortic distensibility; RVEDV, right ventricular end-diastolic volume; RVSV, right ventricular stroke volume; ASI, arterial stiffness index.

^a^AoD and RVEDV have been log-transformed to reduce heteroscedasticity; results are presented as percentage change for these variables.

### Sexual abuse

A history of childhood sexual abuse was reported by 2746 (8.9%) participants, and the rate was double in women than in men (11.7% vs. 5.8%, *P*-value < 0.001, Supplementary data online, *Table S1*). Individuals with a history of sexual abuse had a higher prevalence of smoking, diabetes, and hypertension than the whole sample.

In models adjusted for age and sex (see Supplementary data online, *Table S2*), exposure to childhood sexual abuse was linked to higher LVM and greater LV concentricity. These relationships were attenuated in fully adjusted models, but higher aortic compliance (high AoD) trended towards significance (*Table 2*). There was no significant interaction between sex and sexual abuse (see Supplementary data online, *Table S3*).

### Emotional abuse

Amongst the study cohort, 4685 (22.1%) participants reported a history of childhood emotional abuse (felt hated) (*Table 1*), with higher rates in women than men (17.4% vs. 12.7%, *P*-value < 0.001, Supplementary data online, *Table S1*). Individuals with emotional abuse history had higher rates of smoking and diabetes than the whole sample.

When adjusting for age and sex, a history of emotional abuse was linked to greater LVM, greater LV concentricity, and poorer LV function by LVGFI (see Supplementary data online, *Table S2*). In fully adjusted models, emotional abuse was associated with smaller left and right ventricular volumes (lower LVEDV and lower RVEDV), greater LV concentricity, and poorer function by stroke volume (lower LVSV and lower RVSV) (*Table 2*). Higher myocardial native T1 trended towards significance. There was no significant interaction between sex and emotional abuse (see Supplementary data online, *Table S3*).

### Emotional neglect

There were 6822 (22.1%) participants with a history of childhood emotional neglect (‘felt loved’, *Table 1*), with women reporting at higher rates than men (23.0% vs. 21.2%, *P*-value < 0.001, Supplementary data online, *Table S1*). Individuals with emotional neglect had a greater prevalence of cardiometabolic diseases than the whole cohort.

In age and sex-adjusted models, emotional neglect was linked to smaller right ventricular volume in end-diastole (lower RVEDV) and greater LV concentricity (see Supplementary data online, *Table S2*). In fully adjusted models, the association with greater LV concentricity remained robust (*Table 2*), and smaller LVEDV and RVEDV trended towards significance. There was no significant interaction between sex and emotional neglect (see Supplementary data online, *Table S3*).

### Physical neglect

Amongst the whole sample, childhood physical neglect (someone available to take the child to the doctor) was reported by 14.7% (*n* = 4534) of participants (*Table 1*). Women reported physical neglect at higher rates than men (15.7% vs. 13.6%, *P*-value < 0.001, Supplementary data online, *Table S1*). Participants who experienced physical neglect had the highest prevalence of diabetes, hypertension, and hypercholesterolaemia and the lowest rate of formal educational qualifications compared with the whole cohort and other types of abuse.

Physical neglect was linked to the most prominent CMR remodelling patterns than all the types of adverse experiences considered, and these relationships appeared independent of demographic and cardiometabolic mediators. In models adjusted for age and sex (see Supplementary data online, *Table S2*), exposure to childhood physical neglect was linked to poorer LV function (lower LVSV and lower LVGFI), poorer RV function (lower RVSV), greater LV concentricity, and lower aortic compliance (lower AoD). The associations between physical neglect and adverse LV and RV structure and function (smaller volumes and lower stroke volumes) remained statistically significant in models additionally adjusted for cardiometabolic and demographic factors (*Table 2*). Lower LVGFI trended towards significance. There was no significant interaction between sex and physical neglect (see Supplementary data online, *Table S3*).

## Discussion

In this observational study of 30 814 middle-aged adults from the UK Biobank, we examined associations of ACEs (physical abuse, sexual abuse, emotional abuse, emotional neglect, and physical neglect) with cardiovascular imaging phenotypes. Collectively, all adversity types considered in this study were linked to unhealthy CMR phenotypes, comprising smaller left and right ventricular volumes, greater LVM and a concentric pattern of LV remodelling, poorer LV and RV function, lower aortic compliance, and greater arterial stiffness. No single type of adversity was linked to all CMR phenotypes.

Individuals with adverse childhood exposures had a poorer cardiometabolic profile than the whole sample, most notable in the subset with a history of physical neglect, who also had the most prominent pattern of adverse cardiovascular remodelling than other abuse exposures. For all types of adversity except sexual abuse, many associations remained statistically significant even after adjustment for demographic, lifestyle, and cardiometabolic factors. These observations suggest that although a greater burden of cardiometabolic diseases and unhealthy lifestyles are important mediators of the relationship between childhood adversity and unhealthy CMR phenotypes, these factors do not fully explain the observed associations, indicating the likely contribution of independent biological pathways. Our findings suggest important associations between childhood maltreatment and adult cardiac structure and function, but further research using longitudinal designs and causal inference is necessary to establish the directionality and potential causal nature of these relationships.

Women reported higher rates of all childhood adversity types except physical abuse. Physical abuse was associated with greater LVM in men, but no other significant sex interactions were found for other CMR metrics. Sex differences in associations between childhood adversity and cardiac outcomes have resulted in inconsistent findings.^6,8,11,26,32^ Differences in mediators and pathways linking childhood adversity to CVD are hypothesized to explain this difference.^32^

Our findings add evidence to global literature demonstrating the association between childhood adversity and negative cardiac health in adults in the UK Biobank and in other cohorts.^4,5,8,10,26,32–35^ Three prior studies have looked at the association between childhood maltreatment and CVD in the UK Biobank.^26,32,36^ One of these found all individual types of child maltreatment (except for physical neglect) associated with a higher risk of incident CVD, with a dose–response relationship based on the number of child maltreatment types.^36^ The second study found that all types of maltreatment were associated with an increased risk of CVD and ischaemic heart disease.^26^ The third study assessed the role of mediators between childhood maltreatment and CVD, showing that anxiety/depression, smoking, BMI, and inflammation mediated 26–90% of the associations between childhood maltreatment and CVD.^32^ Although based on the same data, our cohort was smaller, including only individuals who had undergone CMR scanning.

While no prior studies have explored the associations between cardiovascular imaging phenotypes and childhood adversity, several studies have found associations in selected cohorts between childhood maltreatment and other measures of subclinical CVD.^37–39^ Two studies with female participants found childhood sexual abuse associated with higher carotid IMT,^37,38^ while another found associations of physical abuse, emotional abuse, and emotional neglect with higher IMT and an additional association of physical abuse with greater carotid plaque.^38^ Another study found higher levels of plasma C-reactive protein and IL-6 in women reporting sexual abuse.^40^ Our findings extend these existing reports by demonstrating the association of a range of adverse childhood exposures with a comprehensive set of cardiovascular phenotypes, characterizing RV and LV structure and function and arterial health.

Prior studies have linked subclinical CVD and childhood adversity more generally. For example, childhood trauma has been shown to be associated with increased central arterial stiffness,^41^ greater cumulative psychosocial adversity in childhood was associated with greater waist circumference and a lower arterial distensibility,^42^ and adverse childhood family psychosocial environment was associated with greater IMT in white participants.^43^ Childhood abuse and lower parental warmth have also been associated with elevated allostatic load in young and middle adulthood.^44^ Conversely, favourable childhood psychological factors were linked to a lower prevalence of coronary artery calcification in adulthood.^45^ Studies have also explored the impact of cumulative adversity burden. For example, in one study, individuals with four or more ACEs had a greater increase in arterial stiffness from childhood into young adulthood,^46^ while in another, adolescent males aged 10–14 years who experienced four or more ACEs had higher systemic arterial stiffness.^22^

Our results suggest that different types of child maltreatment could impact cardiac function and structure differently. While all types of adverse experiences were linked to unhealthy cardiovascular differences, some showed more pronounced relationships. We found the highest rates of cardiometabolic morbidities and the most prominent adverse remodelling patterns in individuals who experienced childhood physical neglect. Individuals who reported childhood emotional abuse had a similarly (but less profound) adverse cardiometabolic and remodelling profile. Differential effect sizes across different types of maltreatment are a finding that is consistent with prior studies that examined associations with subclinical CVD^38,39^ and CVD outcomes.^47^ While this may suggest that some adverse experiences are more damaging than others, it is challenging to make any such distinctions. Many such experiences likely overlap, leading to disadvantages across multiple areas of health throughout life.

Our findings reinforce the importance of considering childhood adversity as a risk factor not modifiable at the individual level when assessing CVD risk.^8,11,48^ Raising societal awareness of the long-term harms of childhood adversities and steps to reduce these may also improve population health and act as a mechanism for CVD prevention. Potential approaches include public health campaigns by cardiovascular organizations, screening children for exposure to adversities, and identifying those at the highest risk of developing adverse outcomes later in life.^48–50^ Healthcare professionals, such as general practitioners, paediatricians, and cardiologists, who have the most direct patient and parent contact, are well-positioned to mitigate the CVD burden stemming from childhood adversity through clinician education and training on recognizing and addressing the consequences of these experiences.^48^

### Study limitations

This cross-sectional study presents associations, not causal relationships, between childhood experiences and adult cardiac function and structure. The precise timing, duration, and severity of childhood maltreatment experiences were not available, which could influence the strength and nature of associations with adult cardiac outcomes.

Although we adjusted for established cardiovascular risk factors, we cannot exclude the possibility of residual confounding from unmeasured or imprecisely measured factors. Childhood maltreatment and its potential links with adverse adult outcomes are influenced by a complex interplay of social, psychological, and biological factors, which may not be fully accounted for by the covariates in our study.^10,11^ For instance, we were unable to account for all aspects of socioeconomic status throughout the life course, detailed family history of CVD, or specific psychological factors like resilience or coping mechanisms.

The exposure to childhood maltreatment was self-reported by participants, which is subject to self-selection and recall bias. The sensitive nature of the questions may also have negatively influenced the willingness to disclose childhood abuse. In the UK Biobank, childhood maltreatment may be under-reported due to the narrow definitions provided in the questions and the lack of specific examples of what constitutes specific types of abuse. For example, physical neglect includes more than not having someone available to take the child to the doctor. These factors may have reduced the strength of our findings, as the true associations may be higher. This may be particularly true of the observed relationships with sexual abuse, where we had the smallest sample size.

Negative and positive psychological factors are linked with CVD outcomes and have been proposed as a separate category from ACEs for determining cardiovascular risk.^48^ This study focused on the incremental value of the ACEs category to CVD risk prediction over traditional risk factors.

## Conclusion

Individuals with adverse emotional and physical experiences in childhood have poorer cardiometabolic profiles and adverse cardiovascular remodelling patterns in adulthood. Our results highlight the importance of greater awareness and education amongst parents, health professionals, and adults who experienced ACEs about the links to poor cardiovascular outcomes. Clinicians should consider the addition of adverse early life experiences in cardiovascular risk stratification. Future work should explore the use of brain MRI data and CMR metrics from the UK Biobank to investigate neuro-cardiological interactions from childhood adversity, potentially revealing pathways between the potential brain structural or functional changes and the observed cardiovascular remodelling patterns.

## Supplementary Material

qyae139_Supplementary_Data

## Data Availability

This study was conducted within UK Biobank Access Application 2964. The UK Biobank will make the data available to all bona fide researchers for health-related research that is in the public interest. All researchers will be subject to the same application process and approval criteria as specified by the UK Biobank. See the UK Biobank website for more details: http://www.ukbiobank.ac.uk/register-apply/.

## Lead author biography

Juan C. Quiroz is a research fellow at the Centre for Big Data Research in Health at the University of New South Wales, Australia. He obtained his PhD in computer science and engineering from the University of Nevada, Reno (2010). His research focuses on machine learning for healthcare applications, including building prognostic models, developing deep learning survival models, and using large language models with medical records. He has worked on projects predicting cardiovascular and mental health events, identifying patient trajectories, and building medical foundation models.
