# Supplementary material for: The association between adverse childhood experiences and adult cardiac function in the UK Biobank
Source: Eur Heart J Imaging Methods Pract. 2024 Dec 19;2(3):qyae139. doi: 10.1093/ehjimp/qyae139 (PMC11686440; doi:10.1093/ehjimp/qyae139)
Supplement: qyae139_Supplementary_Data [file qyae139_supplementary_data.zip › Supplemental Material.docx]

**Supplemental Materials**

**Binary coding of adverse childhood experiences**

The exposures of interest were the self-reported answers to questions on traumatic experiences during childhood, as part of the UK Biobank online follow-up questions on thoughts and feelings: felt hated by family member as a child (emotional abuse, data-field 20487), physically abused by family as a child (physical abuse, data-field 20488), felt loved as a child (emotional neglect, data-field 20489), sexually molested as a child (sexual abuse, data-field 20490), and someone available to take the child to doctor (physical neglect, data-field 20491). The responses to these questions were coded as an ordinal variable on a 5-point Likert scale, ranging from never true to very often true: 0: never true; 1: rarely true; 2: sometimes true; 3: often true; 4: very often true. These responses were converted to binary for our analysis (threshold values in Table 1), indicating whether the participant experienced the event, consistent with a prior study that analysed the association between adverse childhood experiences (ACEs) and cardiovascular outcomes in the UK Biobank (1,2). Using a binary coding system aligns with numerous studies on ACEs in the UK Biobank (3–6). This approach also allows for easier comparison with existing literature and simplifies the interpretation of results. The rationale for each threshold is as follows:

- Emotional abuse, physical abuse, and sexual abuse: For these ACEs, we set the threshold at "rarely true" or above (≥1 on the Likert scale). This conservative approach ensures that even infrequent occurrences of abuse are captured, acknowledging the potential impact of any abusive experience on long-term health outcomes.
- Emotional neglect: The threshold for this ACE was set at "sometimes true" or below (≤2 on the Likert scale) for the question "felt loved as a child." This threshold identifies individuals who consistently or frequently experienced a lack of emotional support during childhood.
- Physical neglect: This threshold was set at "often true" or below (≤3 on the Likert scale) for the question about someone being available to take the child to the doctor. This threshold captures cases where access to medical care was inconsistent or limited.

**Supplementary Table 1. Threshold values for converting the traumatic experiences responses to binary**

| Adversity | Traumatic experience question | Binary threshold |
| --- | --- | --- |
| Physical abuse | People in my family hit me so hard that it left me with bruises or marks | ≥ 1 |
| Sexual abuse | Someone molested me (sexually) | ≥ 1 |
| Emotional abuse | I felt that someone in my family hated me | ≥ 1 |
| Emotional neglect | I felt loved | ≤ 2 |
| Physical neglect | There was someone to take me to the doctor if I needed it | ≤ 3 |

Responses: 0: never true; 1: rarely true; 2: sometimes true; 3: often true; 4: very often true.

**Image acquisition and analysis**

The UK Biobank Imaging study includes CMR performed in dedicated imaging centres using uniform equipment (1.5 Tesla scanner, MAGNETOM Aera, Siemens, Munich, Germany) staff training, and acquisition protocols (7). Ventricular function was assessed using standard long axis images and a short axis stack covering both ventricles from base to apex. Myocardial tissue character was assessed using myocardial native T1 mapping in one mid-ventricular short axis slice using the ShMOLLI technique (8). Arterial compliance was estimated using aortic distensibility measured from transverse cuts of the thoracic aorta. Image-derived metrics were extracted from scans using fully-automated analysis pipelines previously developed and validated in the UK Biobank (9–11).

**References**

1. Soares ALG, Hammerton G, Howe LD, Rich-Edwards J, Halligan S, Fraser A. Sex differences in the association between childhood maltreatment and cardiovascular disease in the UK Biobank. Heart. 2020 Sep 1;106(17):1310–6.

2. Soares AG, Howe LD, Heron J, Hammerton G, Rich-Edwards J, Magnus MC, et al. How does childhood maltreatment influence cardiovascular disease? A sequential causal mediation analysis. Int J Epidemiol. 2022 Apr 1;51(2):555–66.

3. Hanlon P, McCallum M, Jani BD, McQueenie R, Lee D, Mair FS. Association between childhood maltreatment and the prevalence and complexity of multimorbidity: A cross-sectional analysis of 157,357 UK Biobank participants. J Comorbidity. 2020 Jan 1;10:2235042X10944344.

4. Chaplin AB, Jones PB, Khandaker GM. Sexual and physical abuse and depressive symptoms in the UK Biobank. BMC Psychiatry. 2021 May 11;21(1):248.

5. Cheng S, Wen Y, Liu L, Cheng B, Liang C, Ye J, et al. Traumatic events during childhood and its risks to substance use in adulthood: an observational and genome-wide by environment interaction study in UK Biobank. Transl Psychiatry. 2021 Aug 20;11(1):1–6.

6. Gheorghe DA, Li C, Gallacher J, Bauermeister S. Associations of perceived adverse lifetime experiences with brain structure in UK Biobank participants. J Child Psychol Psychiatry. 2021;62(7):822–30.

7. Petersen SE, Matthews PM, Francis JM, Robson MD, Zemrak F, Boubertakh R, et al. UK Biobank’s cardiovascular magnetic resonance protocol. J Cardiovasc Magn Reson. 2016 Feb 1;18(1):8.

8. Piechnik SK, Ferreira VM, Dall’Armellina E, Cochlin LE, Greiser A, Neubauer S, et al. Shortened Modified Look-Locker Inversion recovery (ShMOLLI) for clinical myocardial T1-mapping at 1.5 and 3 T within a 9 heartbeat breathhold. J Cardiovasc Magn Reson. 2010 Nov 19;12(1):69.

9. Bai W, Sinclair M, Tarroni G, Oktay O, Rajchl M, Vaillant G, et al. Automated cardiovascular magnetic resonance image analysis with fully convolutional networks. J Cardiovasc Magn Reson. 2018 Sep 14;20(1):65.

10. Hann E, Popescu IA, Zhang Q, Gonzales RA, Barutçu A, Neubauer S, et al. Deep neural network ensemble for on-the-fly quality control-driven segmentation of cardiac MRI T1 mapping. Med Image Anal. 2021 Jul 1;71:102029.

11. Biasiolli L, Hann E, Lukaschuk E, Carapella V, Paiva JM, Aung N, et al. Automated localization and quality control of the aorta in cine CMR can significantly accelerate processing of the UK Biobank population data. PLOS ONE. 2019 Feb 14;14(2):e0212272.
