# Supplementary material for: The association between adverse childhood experiences and adult cardiac function in the UK Biobank
Source: Eur Heart J Imaging Methods Pract. 2024 Dec 19;2(3):qyae139. doi: 10.1093/ehjimp/qyae139 (PMC11686440; doi:10.1093/ehjimp/qyae139)
Supplement: qyae139_Supplementary_Data [file qyae139_supplementary_data.zip › Supplemental Table 1.docx]

**Supplemental Table 1: Tests for sex differences in rates of reported childhood maltreatment**

| **Group** | **Male cases** (out of 14,386) | **Female cases** (out of 16,428) | **Male %** | **95% CI for  Male %** | **Female %** | **95 % CI for  Female %** | **p-value** |
| --- | --- | --- | --- | --- | --- | --- | --- |
| Physical abuse | 3,062 | 2,961 | 21.3% | [20,6, 22.0] | 18.0% | [17.4, 18.6] | < 0.001 |
| Sexual abuse | 831 | 1,915 | 5.8% | [5.4, 6.2] | 11.7% | [11.2, 12.2] | < 0.001 |
| Emotional neglect | 3,049 | 3,773 | 21.2% | [20.5, 21.9] | 23.0% | [22.3, 23.6] | < 0.001 |
| Emotional abuse | 1,825 | 2,860 | 12.7% | [12.1, 13.2] | 17.4% | [16.8, 18.0] | < 0.001 |
| Physical neglect | 1,963 | 2,571 | 13.6% | [13.1, 14.2] | 15.7% | [15.1, 16.2] | < 0.001 |

Using two-sample Z-tests for equality of proportions with continuity correction, with a two-sided p-value.
