# Supplementary material for: The association between adverse childhood experiences and adult cardiac function in the UK Biobank
Source: Eur Heart J Imaging Methods Pract. 2024 Dec 19;2(3):qyae139. doi: 10.1093/ehjimp/qyae139 (PMC11686440; doi:10.1093/ehjimp/qyae139)
Supplement: qyae139_Supplementary_Data [file qyae139_supplementary_data.zip › Supplemental Table 2.docx]

**Supplemental Table 2. Associations of adverse childhood experiences with CMR metrics**

|  | **Physical abuse** | | **Sexual abuse** | | **Emotional neglect** | | **Emotional abuse** | | **Physical neglect** | |
| --- | --- | --- | --- | --- | --- | --- | --- | --- | --- | --- |
|  | Minimally adjusted | Fully adjusted | Minimally adjusted | Fully adjusted | Minimally adjusted | Fully adjusted | Minimally adjusted | Fully adjusted | Minimally adjusted | Fully adjusted |
| LVEDV (ml) | ***1.153***  ***(0.404, 1.903)*** | 0.101  (-0.685, 0.886) | -0.070  (-1.115, 0.975) | -0.356  (-1.463, 0.751) | -0.719  (-1.432, -0.006) | -0.921  (-1.678, -0.164) | -0.392  (-1.222, 0.437) | ***-1.210***  ***(-2.085, -0.335)*** | -0.711  (-1.550, 0.127) | ***-1.216***  ***(-2.113, -0.318)*** |
| P-value  q-value | ***0.003***  ***0.011*** | 0.802  0.882 | 0.895  0.947 | 0.529  0.677 | 0.048  0.115 | 0.017  0.067 | 0.354  0.513 | ***0.007***  ***0.041*** | 0.096  *0.201* | ***0.008***  ***0.042*** |
| LVSV (ml) | 0.519  (0.054, 0.984) | -0.015  (-0.503, 0.474) | 0.141  (-0.789, 0.508) | -0.327  (-1.015, 0.361) | -0.364  (-0.806, 0.079) | -0.363  (-0.834, 0.108) | -0.348  (-0.862, 0.167) | ***-0.827***  ***(-1.371, -0.283)*** | ***-0.683***  ***(-1.203, -0.162)*** | ***-1.013***  ***(-1.571, -0.455)*** |
| P-value  q-value | 0.029  0.075 | 0.953  0.960 | 0.670  0.802 | 0.352  0.569 | 0.107  0.201 | 0.131  0.279 | 0.185  0.309 | ***0.003***  ***0.028*** | ***0.010***  ***0.03*** | ***< 0.001***  ***0.005*** |
| LVM (g) | ***1.854***  ***(1.417, 2.290)*** | 0.538  (0.114, 0.963) | ***0.811***  ***(0.202, 1.420)*** | -0.142  (-0.740, 0.457) | 0.477  (0.061, 0.892) | -0.029  (-0.438, 0.380) | ***1.099***  ***(0.616, 1.582)*** | -0.012  (-0.485, 0.461) | 0.286  (-0.203, 0.775) | -0.480  (-0.966, 0.005) |
| P-value  q-value | ***< 0.001***  ***<0.001*** | 0.013  0.055 | ***0.009***  ***0.029*** | 0.643  *0.804* | 0.025  0.068 | 0.890  0.941 | ***< 0.001***  ***<0.001*** | 0.960  0.960 | 0.252  0.396 | 0.052  0.170 |
| LVM: LVEDV (g/ml) | ***0.008***  ***(0.006, 0.011)*** | ***0.004***  ***(0.001, 0.006)*** | ***0.006***  ***(0.003, 0.009)*** | 0.001  (-0.003, 0.004) | ***0.006***  ***(0.004, 0.009)*** | ***0.003***  ***(0.001, 0.006)*** | ***0.009***  ***(0.007, 0.012)*** | ***-0.012***  ***(0.002, 0.008)*** | ***0.006***  ***(0.003, 0.008)*** | 0.002  (0.000, 0.005) |
| P-value  q-value | ***< 0.001***  ***<0.001*** | ***0.003***  ***0.028*** | ***< 0.001***  ***0.001*** | 0.678  *0.829* | ***< 0.001***  **<0.001** | ***0.004***  ***0.031*** | ***< 0.001***  ***<0.001*** | ***0.0003***  ***0.028*** | ***< 0.001***  ***<0.001*** | 0.097  0.249 |
| LV GLS (%) | 0.043  (-0.054, 0.139) | 0.050  (-0.053, 0.154) | -0.002  (-0.138, 0.134) | 0.063  (-0.084, 0.211) | 0.008  (-0.085, 0.100) | 0.016  (-0.083, 0.116) | 0.001  (-0.106, 0.108) | 0.050  (-0.065, 0.165) | -0.037  (-0.146, 0.072) | -0.018  (-0.137, 0.100) |
| P-value  q-value | 0.387  0.532 | 0.341  0.569 | 0.973  0.985 | 0.400  0.595 | 0.871  0.939 | 0.750  0.874 | 0.985  0.985 | 0.394  0.595 | 0.503  0.629 | 0.763  0.874 |
| LVGFI (%) | ***-0.005***  ***(-0.007, -0.003)*** | -0.002  (-0.004, 0.000) | -0.003  (-0.006, 0.000) | -0.001  (-0.004, 0.002) | -0.002  (-0.004, 0.000) | 0.000  (-0.002, 0.002) | ***-0.004***  ***(-0.007, -0.002)*** | -0.002  (-0.005, 0.000) | ***-0.003***  ***(-0.006, -0.001)*** | -0.003  (-0.005, 0.000) |
| P-value  q-value | ***< 0.001***  ***<0.001*** | 0.059  0.179 | 0.048  0.115 | 0.447  0.615 | 0.103  0.201 | 0.802  0.882 | ***< 0.001***  ***<0.001*** | 0.062  0.179 | ***0.004***  ***0.015*** | 0.029  0.099 |
| T1 (ms) | -0.085  (-1.060, 0.891) | 0.814  (-0.220, 1.848) | -0.553  (-1.915, 0.810) | 0.048  (-1.412, 1.508) | 1.116  (0.184, 2.047) | 0.812  (-0.187, 1.812) | 0.987  (-0.096, 2.069) | 1.378  (0.224, 2.532) | 0.691  (-0.404, 1.787) | 0.997  (-0.189, 2.183) |
| P-value  q-value | 0.865  0.939 | 0.123  0.279 | 0.427  0.572 | 0.949  0.960 | 0.019  0.055 | 0.111  0.265 | 0.074  0.163 | 0.019  0.071 | 0.216  *0.349* | 0.099  0.249 |
| AoD*  (x10-3 mmHg) | 0.420  (-1.282, 2.153) | 1.363  (-0.475, 3.236) | 1.260  (-1.153, 3.733) | 2.484  (-0.156, 5.193) | -1.186  (-2.784, 0.438) | -0.958  (-2.690, 0.804) | -0.289  (-2.161, 1.619) | 0.702  (-1.333, 2.779) | ***-2.532***  ***(-4.383, -0.646)*** | -1.233  (-3.283, 0.860) |
| P-value  q-value | 0.631  0.771 | 0.147  0.299 | 0.309  0.459 | 0.065  0.179 | 0.151  0.260 | 0.284  0.521 | 0.765  0.859 | 0.502  0.657 | ***0.009***  ***0.029*** | 0.246  0.478 |
| ASI (m/s) | 0.145  (0.056, 0.234) | 0.055  (-0.040, 0.150) | 0.006  (-0.120, 0.131) | -0.070  (-0.205, 0.065) | 0.033  (-0.052, 0.118) | -0.044  (-0.135, 0.048) | 0.036  (-0.063, 0.135) | -0.046  (-0.152, 0.061) | 0.081  (-0.018, 0.181) | 0.012  (-0.096, 0.120) |
| P-value  q-value | **0.001**  **0.006** | 0.252  0.478 | 0.930  0.965 | 0.310  0.550 | 0.450  0.589 | 0.352  0.569 | 0.475  0.607 | 0.400  0.595 | 0.110  0.201 | 0.826  0.891 |
| RVEDV* (ml) | 0.411  (-0.091, 0.915) | -0.214  (-0.731, 0.305) | -0.125  (-0.819, 0.574) | -0.295  (-1.021, 0.437) | ***-0.638***  ***(-1.110, -0.163)*** | -0.638  (-1.134, -0.140) | -0.253  (-0.804, 0.301) | ***-0.808***  ***(-1.380, -0.232)*** | ***-1.290***  ***(-1.841, -0.736)*** | ***-1.326***  ***(-1.910, -0.739)*** |
| P-value  q-value | 0.109  0.201 | 0.418  0.605 | 0.726  0.849 | 0.429  0.605 | ***0.009***  **0.029** | 0.012  0.055 | 0.370  0.522 | ***0.006***  ***0.041*** | ***< 0.001***  ***<0.001*** | ***< 0.001***  ***<0.001*** |
| RVSV (ml) | 0.358  (-0.125, 0.840) | -0.099  (-0.607, 0.409) | -0.105  (-0.777, 0.568) | -0.268  (-0.983, 0.448) | -0.446  (-0.906, 0.013) | -0.376  (-0.866, 0.113) | -0.301  (-0.835, 0.233) | ***-0.762***  ***(-1.328, -0.196)*** | ***-1.075***  ***(-1.615, -0.536)*** | ***-1.254***  ***(-1.910, -0.739)*** |
| P-value  q-value | 0.146  0.259 | 0.703  0.841 | 0.761  0.859 | 0.463  0.621 | 0.057  0.130 | 0.132  0.279 | 0.269  0.410 | ***0.008***  ***0.042*** | ***< 0.001***  ***<0.001*** | ***< 0.001***  ***<0.001*** |

**Supplemental Table 2 footnote.** Results are absolute change in CMR metric expressed as Beta (95% confidence interval). The p-value and false discovery rate q-value are included below the Beta estimates. The association of each exposure (binary Yes/No) with CMR metrics was estimated with linear regression models. Minimally adjusted models were adjusted for age and sex. Fully adjusted models were adjusted for age, sex, body mass index, Townsend deprivation score, smoking, diabetes, hypertension, hypercholesterolaemia, alcohol use, exercise, education. *AoD and RVEDV have been log-transformed to reduce heteroscedasticity; results are presented as percentage change for these variables. LVEDV: left ventricular end-diastolic volume; LVSV: left ventricular stroke volume LVM: left ventricular mass; LV GLS: left ventricular global longitudinal strain; LVGFI: left ventricular global functional index; AoD: aortic distensibility; RVEDV: right ventricular end-diastolic volume; RVSV: right ventricular stroke volume; ASI: arterial stiffness index.
