# Supplementary material for: The association between adverse childhood experiences and adult cardiac function in the UK Biobank
Source: Eur Heart J Imaging Methods Pract. 2024 Dec 19;2(3):qyae139. doi: 10.1093/ehjimp/qyae139 (PMC11686440; doi:10.1093/ehjimp/qyae139)
Supplement: qyae139_Supplementary_Data [file qyae139_supplementary_data.zip › Supplemental Table 3.docx]

**Supplemental Table 3. Interaction effects between CMR metrics and sex for adverse childhood experiences in fully adjusted models.**

|  | **Physical abuse** | **Sexual abuse** | **Emotional neglect** | **Emotional abuse** | **Physical neglect** |
| --- | --- | --- | --- | --- | --- |
| **LVEDV (ml)** | 1.440  (-0.120, 3.000) | 1.960  (-0.390, 4.320) | -0.390  (-1.900, 1.120) | -0.790  (-2.550, 0.970) | -0.130  (-1.910, 1.660) |
|  | p=0.071  q=0.362 | p=0.102  q=0.424 | p=0.612  q=0.820 | p=0.378  q=0.676 | p=0.888  q=0.958 |
| **LVSV (ml)** | 0.870  (-0.100, 1.840) | 0.720  (-0.750, 2.180) | -0.240  (-1.180, 0.700) | -0.580  (-1.680, 0.510) | -0.160  (-1.270, 0.950) |
|  | p=0.079  q=0.362 | p=0.336  q=0.660 | p=0.620  q=0.820 | p=0.296  q=0.615 | p=0.781  q=0.914 |
| **LVM (g)** | **2.120  (1.280, 2.960)** | 1.340  (0.060, 2.610) | 0.280  (-0.530, 1.100) | 0.640  (-0.310, 1.590) | -0.070  (-1.040, 0.900) |
|  | **p < 0.001**  **q < 0.001** | p=0.040  q=0.362 | p=0.499  q=0.782 | p=0.189  q=0.520 | p=0.888  q=0.958 |
| **LVM: LVEDV**  **(g/ml)** | 0.007  (0.002, 0.011) | -0.001  (-0.008, 0.006) | 0.001  (-0.003, 0.006) | 0.005  (0.000, 0.010) | -0.001  (-0.007, 0.004) |
|  | p=0.006  q=0.110 | p=0.748  q=0.894 | p=0.564  q=0.816 | p=0.074  q=0.362 | p=0.667  q=0.834 |
| **LV GLS (%)** | 0.110  (-0.100, 0.310) | 0.040  (-0.270, 0.350) | -0.060  (-0.260, 0.130) | 0.100  (-0.130, 0.330) | 0.000  (-0.240, 0.230) |
|  | p=0.302  q=0.615 | p=0.801  q=0.918 | p=0.526  q=0.782 | p=0.381  q=0.676 | p=0.992  q=0.992 |
| **LVGFI (%)** | -0.004  (-0.008, 0.001) | -0.003  (-0.009, 0.004) | 0.000  (-0.004, 0.004) | -0.001  (-0.006, 0.004) | 0.004  (-0.001, 0.009) |
|  | p=0.116  q=0.425 | p=0.449  q=0.748 | p=0.915  q=0.960 | p=0.626  q=0.820 | p=0.144  q=0.495 |
| **T1 (ms)** | -2.940  (-4.990, -0.880) | 1.810  (-1.290, 4.910) | -2.020  (-4.010, -0.020) | -1.610  (-3.930, 0.700) | 2.420  (0.060, 4.780) |
|  | p=0.005  q=0.110 | p=0.253  q=0.605 | p=0.048  q=0.362 | p=0.173  q=0.504 | p=0.045  q=0.362 |
| **AoD***  **(x10^-3^ mmHg)** | -3.500  (-6.950, 0.070) | -3.370  (-8.570, 2.130) | -2.490  (-5.850, 1.000) | -2.300  (-6.210, 1.780) | 1.550  (-2.590, 5.870) |
|  | p=0.055  q=0.362 | p=0.225  q=0.563 | p=0.160  q=0.504 | p=0.265  q=0.607 | p=0.469  q=0.759 |
| **ASI (m/s)** | 0.010  (-0.180, 0.200) | 0.260  (-0.030, 0.550) | 0.060  (-0.120, 0.240) | -0.020  (-0.230, 0.200) | 0.060  (-0.160, 0.270) |
|  | p=0.940  q=0.960 | p=0.075  q=0.362 | p=0.516  q=0.782 | p=0.880  q=0.958 | p=0.607  q=0.820 |
| **RVEDV* (ml)** | 0.720  (-0.310, 1.760) | 1.760  (0.190, 3.360) | 0.040  (-0.960, 1.040) | -0.530  (-1.680, 0.630) | 0.480  (-0.700, 1.670) |
|  | p=0.174  q=0.504 | p=0.028  q=0.362 | p=0.943  q=0.960 | p=0.369  q=0.676 | p=0.428  q=0.736 |
| **RVSV (ml)** | 0.830  (-0.180, 1.830) | 0.950  (-0.570, 2.470) | -0.220  (-1.200, 0.760) | -0.600  (-1.740, 0.540) | -0.190  (-1.350, 0.960) |
|  | p=0.108  q=0.424 | p=0.221  q=0.563 | p=0.659  q=0.834 | p=0.300  q=0.615 | p=0.743  q=0.894 |

**Table 2 footnote.** Models adjusted for age, sex, body mass index, Townsend deprivation score, smoking, diabetes, hypertension, hypercholesterolemia, alcohol use, exercise, education expressed as Beta (95% confidence interval). P-value and q-value (false discovery rate) are included below the Beta estimates. *AoD and RVEDV have been log-transformed to reduce heteroscedasticity; results are presented as percentage change for these variables. LVEDV: left ventricular end-diastolic volume; LVSV: left ventricular stroke volume LVM: left ventricular mass; LV GLS: left ventricular global longitudinal strain; LVGFI: left ventricular global functional index; AoD: aortic distensibility; RVEDV: right ventricular end-diastolic volume; RVSV: right ventricular stroke volume; ASI: arterial stiffness index.
